# Carbon Partitioning in Green Algae (Chlorophyta) and the Enolase Enzyme

**DOI:** 10.3390/metabo4030612

**Published:** 2014-08-04

**Authors:** Jürgen E. W. Polle, Peter Neofotis, Andy Huang, William Chang, Kiran Sury, Eliza M. Wiech

**Affiliations:** 1 Department of Biology, Brooklyn College of the City University of New York, 2900 Bedford Avenue 200NE, Brooklyn, NY 11210, USA; E-Mails: pneofotis@gc.cuny.edu (P.N.); gnauhandy@gmail.com (A.H.); williamchang3389@gmail.com (W.C.); kiran_sury92@yahoo.com (K.S.); EWiech@gc.cuny.edu (E.M.W.); 2 The Graduate Center of the City University of New York, 2900 Bedford Avenue 200NE, Brooklyn, NY 11210, USA

**Keywords:** green algae, metabolism, carbon partitioning, enolase

## Abstract

The exact mechanisms underlying the distribution of fixed carbon within photoautotrophic cells, also referred to as carbon partitioning, and the subcellular localization of many enzymes involved in carbon metabolism are still unknown. In contrast to the majority of investigated green algae, higher plants have multiple isoforms of the glycolytic enolase enzyme, which are differentially regulated in higher plants. Here we report on the number of gene copies coding for the enolase in several genomes of species spanning the major classes of green algae. Our genomic analysis of several green algae revealed the presence of only one gene coding for a glycolytic enolase [EC 4.2.1.11]. Our predicted cytosolic localization would require export of organic carbon from the plastid to provide substrate for the enolase and subsequent re-import of organic carbon back into the plastids. Further, our comparative sequence study of the enolase and its 3D-structure prediction may suggest that the N-terminal extension found in green algal enolases could be involved in regulation of the enolase activity. In summary, we propose that the enolase represents one of the crucial regulatory bottlenecks in carbon partitioning in green algae.

## 1. Introduction

In the past few years algal biofuels have received renewed attention [1,2,3]. The term “alga” does not refer to a monophyletic group of organisms, but rather encompasses a large number of organisms from a variety of different origins, including sometimes multiple endosymbiotic events, resulting in multiple lineages of organisms [4,5]. All algae perform oxygenic photosynthesis, but the varieties of lineages have significantly different cellular organizations and diversity in their capabilities to synthesize and store metabolites. Here, we focus on the green algae (Chlorophyta) that are part of the “green lineage” and are believed to have arisen through secondary endosymbiosis [4,5,6]. Currently, the green algae are grouped into four core chlorophyte classes (Chlorophyceae, Trebouxiophyceae, Ulvophyceae, Chlorodendrophyceae) and the prasinophytes, which consist of multiple clades [6]. In the past, it was believed that green algae were simply single-celled plants. This view, however, was recently challenged. For example, one major distinction in metabolism between the green algae and the Streptophytes is the absence of the mevalonate pathway for isoprenoid biosynthesis in the green algae [7].

**Figure 1:**
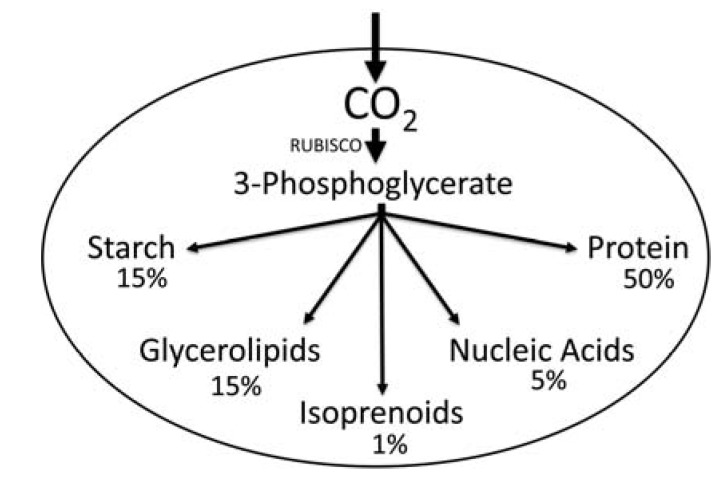
General overview of carbon partitioning with approximate chemical composition in % of dry weight in a green algal cell [9]. Note that the amounts of the different chemicals, such as protein, starch, and lipid, are highly variable depending on the growth conditions and species.

Figure 1 shows a general overview of the path of carbon leading from fixation of CO_2_ to the different major cellular components, following referred to as “carbon partitioning”, in a phototrophically grown green algal cell. Carbon partitioning in cells involves multiple pathways together representing a carbon metabolic network. In algae, the core carbon metabolism includes the plastid localized Calvin-Benson cycle (=Reductive Pentose Phosphate Pathway). In the light, operation of the Calvin-Benson cycle “begins” with fixation of CO_2_ through the enzyme Ribulose-1,5-bisphosphate carboxylase/oxygenase (RUBISCO, EC 4.1.1.39) into 3-phosphoglycerate (3PGA). Then 3PGA can be used for conversion into multiple different cellular macromolecules (Figure 1). In general, following reduction of 3PGA into triose phosphates, condensation of two triose phosphate molecules can create hexose phosphates that may either be used for regeneration of ribulose-1,5-bisphosphate or for the synthesis of starch. Also, either triose phosphates or hexose phosphates could leave the Calvin-Benson cycle to be exported from the plastid for use in other cellular compartments. In the context of the core carbon metabolism, specifically important for cells is the connection of carbon fixed in the Calvin-Benson cycle to create pyruvate, which is needed in cells as a precursor to form a variety of macromolecules including glycerolipids and isoprenoids. This connection between 3PGA to pyruvate synthesis is determined by the reactions of glycolysis/gluconeogenesis, and the cellular localization of the essential enzymes. Depending on the organism, various alternative options for conversion of 3PGA to pyruvate may exist for organisms, but metabolic constraints drive certain possible paths [8].

Understanding of the molecular mechanisms underlying regulation of carbon partitioning is fundamental for genetic engineering and development of novel strains of green algae that for example will have improved growth and lipid productivities. However, currently a major gap in knowledge exists regarding the molecular mechanism regulating carbon partitioning in algal cells, including the green algae. This lack of knowledge may still be a relict from the historical vision of green algae as simply single celled plants. Consequently, we focus here on the contrast between higher plants and green algae.

## 2. Results and Discussion

### 2.1. Carbon Partitioning in Green Algae

Earlier work on unraveling the molecular mechanisms of regulation of carbon partitioning in algae traces back at least to the “Aquatic Species Program” [10] and has been investigated for various microalgae [11,12,13], but a number of issues remain unresolved [14]. For example, it is still unknown if one or two complete glycolytic pathways exist in green algae. In green algal and plant metabolism, phosphoenolpyruvate (PEP) plays an important role–as it is the precursor to pyruvate. In photosynthetic eukaryotes, many of the biosynthetic pathways using pyruvate are localized only in the plastid, and it was reported previously that plants without proper supply of PEP as a precursor for pyruvate to plastids exhibit impaired growth and development [15,16]. An absence of the lower part of glycolyis from the chloroplast and the localization of its enzymes only to the cytosol requires that fixed carbon must be exported by the chloroplast into the cytosol fraction of the cell. There, the glycolytic enolase enzyme could generate PEP, which could then be converted into pyruvate. The cytosolic PEP and/or pyruvate could then be used as a source metabolite for import of carbon back into the plastid [17]. Based on information from higher plants, it would be expected that the presence of enzymes in the plastid allow for carbon flow from the Calvin-Benson cycle directly to pyruvate. This should then make dramatic differences in the distribution of carbon within green algal cells. Unfortunately, even in the best-studied green model alga *Chlamydomonas reinhardtii* the exact path of carbon remains unresolved. For example, some metabolic models contain the enolase activity present in the plastid [18,19], other models have the enolase absent from the plastids [20,21], or compartmentation was not taken into account [22].

For our integrative approach to study carbon partitioning in green algae, we used higher plants such as *Arabidopsis thaliana* for comparison, because much more knowledge exists on carbon metabolism of higher plants [17]. Similar to a previous study [22], based on reference information available from several databases including KEGG and MetaCyc, we created a draft core network for carbon metabolism in green algae. Specifically application of recent “omics” technologies has enabled accumulation of large-scale data sets [23,24] that aided us in extending the draft network by inclusion of published data for *C*. *reinhardtii* [20,21,25,26]. Our focus was on understanding the central carbohydrate metabolism with carbon distribution to glycerolipids and isoprenoids in the cellular compartmentation context in green algae. Such differential cellular compartmentalization of essential reactions of glycolysis/gluconeogenesis was predicted to have major consequences regarding the cellular path of carbon following CO_2_ fixation within green algal cells.

**Figure 2:**
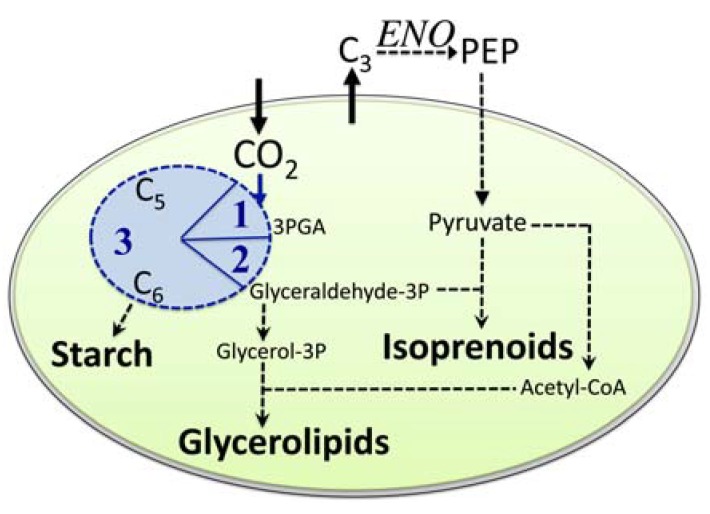
Simplified schematic showing our proposed path for carbon partitioning within the plastid of a green algal cell focused on starch, lipids, and isoprenoids. Note, that in addition to import of CO_2_ into the plastid, also either pyruvate directly or a precursor metabolite needs to be imported that could then be converted into pyruvate. ENO indicates the enolase enzyme outside the plastid. C_3_, C_5_, and C_6_ represent monosaccharides with three, five, or six carbon atoms, respectively. The blue circle represents the Calvin-Benson cycle with 1 = Carbon fixation, 2 = reduction phase, 3 = regeneration phase.

Our analysis of available data [26,27,28,29] strongly suggested that the crucial enzyme enolase is only present in the cytosol of *C*. *reinhardtii*. Consequently, Figure 2 shows a simplified summary of our metabolic network reconstruction results for green algae with proposed major routes of fixed carbon into starch, glycerolipids, and isoprenoids. The carbon flow as proposed in Figure 2 requires carbon exchange between the plastid and cytosol due to the localization of the enolase only to the cytosol. Consequently, the enolase activity might represent a major control point and possible regulator for carbon partitioning in green algae, because the distribution of carbon into macromolecules is not constitutive. As allocation of fixed carbon into different cellular components (e.g., glycerolipids and isoprenoids) is altered by green algae to satisfy the cellular needs during acclimation to changing environments, regulation of carbon partitioning has to occur. One example is *Dunaliella salina*, where cells react to reduced availability of nutrients–for example nitrogen–by switching from primary metabolism geared towards growth towards secondary stress metabolism, which includes over-accumulation of β-carotene in plastidic carotene globules [30]. In non-stressed cells, the amount of β-carotene is less than 0.1% of the total cellular biomass, but reduced availability of nitrogen results in accumulation of β-carotene to a level of more than 8% of the total cellular biomass [30]. Such massive accumulation of β-carotene is due to *de novo* biosynthesis of isoprenoids, which is evidence for massive re-routing of carbon under cellular stress into isoprenoid biosynthesis.

In green algae, isoprenoids are only made through the plastid localized methyl-erythritol-phosphate (MEP) pathway using pyruvate as a precursor molecule. As PEP is a precursor to pyruvate, we hypothesize that the enolase represents a possible bottleneck in carbon partitioning in green algae. Specifically under stress conditions when photosynthesis in algae is uncoupled from growth, the enolase might become a major regulatory point in adjusting carbon partitioning in response to environmental changes.

### 2.2. The Enolase

The enolase is a multi-functional protein [31,32,33]. It operates not only as an essential phosphopyruvate hydratase (EC 4.2.1.11) enzyme in glycolytic catabolism or in anabolic gluconeogenesis converting reversibly 2-phospho-D-glycerate (2PGA) into PEP, but the enolase protein was also reported to have non-glycolytic functions making it a moonlighting protein [32,33,34,35]. For example, the enolase protein was implicated as a cell surface receptor in a wide range of organisms from bacteria [36,37,38] to eukaryotic parasites [39,40,41]. It can be a structural component of animal eyelenses [42], or–as an alternate translation product–a partial enolase protein can operate as a transcriptional repressor [43,44,45]. In addition to the enolase protein having different functions within one cell, an enolase superfamily exists with not all predicted gene products working as enolases. One example is the recent discovery of family members which function as D-mannonate and D-gluconate dehydratases [46].

As a highly conserved enzyme functioning in central carbohydrate metabolism, the glycolytic enolase is an ancient enzyme present in the earliest precursors of all cells found in bacteria, archaea, and all eukaryotes cells. Following, we will refer to the enolase acting as a phosphopyruvate hydratase (EC 4.2.1.11) in glycolysis (the classical Embden-Meyerhof-Parnas Pathway) as the glycolytic enolase. However, it should be noted that according to the KEGG database, the enolase (EC 4.2.1.11) is also annotated as acting alternatively on 3-phospho-d-erythronate to form phosphoenol-4-deoxy-3-tetrulosonate. In general, in eukaryotic cells the enolase is known to form dimers [47]. In vertebrates, the enolase superfamily consists of homo- and hetero-dimers made up of alpha-, beta-, and gamma-isoforms [48,49]. Similarly, in the green alga *D. salina*, the enolase was reported to be present in dimeric form [50].

In our comparative approach to study carbon core metabolism in green algae, we used the known metabolism of higher plants as a basis to expand our understanding. Higher plants such as *A*. *thaliana* have a gene family coding for the enolase enzyme [51], with the gene products being localized to different cellular compartments [52]. Further, it is known that metabolism in higher plants is differentially regulated at the organ level based upon the presence or absence of a family of enolase enzymes. In the model plant *A*. *thaliana*, overall four genes code for enolases:

(1) A plastid-localized enolase (ENO1) [53].
(2) An enolase (ENO2 also called LOS2), which is very similar in sequence and structure to those of animals and yeast [54] and most likely localizes to the cytosol and as an alternative translation product (MBP-1) to the nucleus where it acts as a transcriptional suppressor [44,45].
(3) An enolase localized to the cytosol (ENOc [16]),
(4) A multi-functional enolase (DEP1) [55], which was detected in the chloroplast stroma in *A*. *thaliana* [55]. DEP1 in *Arabidopsis* functions in the methionine biosynthesis [56] performing the reaction of the 5’-methylthioribulose-1-phosphate dehydratase (EC 4.2.1.109) and of the Enolase-phosphatase E1 (EC 3.1.3.77). The DEP1 enolase activity converts 2,3-diketo-5-methylthiopentyl-1-phosphate into the intermediate 2-hydroxy-3-keto-5-methylthiopentenyl-1-phosphate, which is then dephosphorylated. DEP1 is an essential enzyme in cellular sulfur metabolism in plants [57].

As pyruvate is a central precursor metabolite for many different cellular products such as glycerolipids, isoprenoids, and amino acids, the cellular localization and activity of the enolase enzymes may be at the crossroad for carbon partitioning in plant cells. The finding that a high level of expression of enolase was implicated in being necessary for oil accumulation in sunflower seeds supported the importance of enolase in carbon partitioning [58]. Nevertheless, it appears that the plastidal ENO1 is dispensable at certain growth stages, because ENO1 deficient Arabidopsis plants had wild-type amounts of seed oil at maturity [52]. Therefore, a cytosolic enolase activity alone seems to be able to support plant growth, whereas disruption of cytosolic enolase activity has detrimental effects of growth [16].

### 2.3. The Enolase in Green Algae

The glycolytic enolase is the crucial enzyme for cells to create PEP, which can then be processed to generate pyruvate. However, relatively little information is available for the glycolytic enolase of green algae. Until recently, for green algae the number of genes coding for enolase and their cellular distribution was not clear [59]. However, lately several genomes of green algae were sequenced and are now available from online databases, such as of the US Joint Genome Institute (JGI [60]). Here, we investigated the genomes of several species belonging to core chlorophytes, such as *Chlamydomonas reinhardtii* [61], *Volvox carteri* [62], *Asterochloris sp*., *Chlorella variabilis* NC64A [63], and *Coccomyxa subelipsoidea* C-169 [64], as well as prasinophytes represented by *Micromonas pusilla* strains RCC299 and CCMP1545 [65], *Ostreococcus lucimarinus* and *Ostreococcus tauri* [66].

Table 1 summarizes our results of a comparative genomic analysis on the search for gene copies present in genomes obtained from the JGI database. In addition to sequences obtained from the JGI database, for our analysis we included the enolase protein sequence deduced from genomic and transcriptomic data from the unicellular green alga *D. salina* ([60], project #16719 and project #1014865). Similar to *C*. *reinhardtii* and *V*. *carteri*, the genome of *D*. *salina* only contained one enolase. The trebouxiophytes *Asterochloris sp*. and *Chlorella variabilis* NC64A also had only one gene coding for the enolase. In contrast, the trebouxiophyte *C*. *subelipsoidea* C-169 contained two genes coding for identical enolase proteins and further examination of the genomic sequences showed that they were identical as well, indicating a more recent gene duplication event. The prasinophytes *M*. *pusilla* strains RCC299 and CCMP1445 as well as *O*. *lucimarinus* and *O*. *tauri* had each one copy coding for the enolase. In addition to our results with species of *Micromonas* and *Ostreococcus*, the presence of two enolase gene copies had previously been reported for the prasinophyte *Pycnococcus provasolii* [59]. Although the sequences were not complete due to missing N-terminus and C-terminus, the alignment of the two enolase protein sequences of *P*. *provasolii* (Enolase 1-GenBank #AAL05456.1; Enolase 2-GenBank #AAL05457.1) showed that they were not identical (Data not presented) and three insertions found in the enolase 1 of *P*. *provasolii* indicated plasticity within the species. In brief, one or multiple enolase genes could be found in the genomes of green algae.

**Table 1:** Information on the genes coding for the enolase in green algae as extracted from the database of the US Joint Genome Institute (as of 23 May 2014).

Species	JGI Protein ID	JGI Locus Name & Location	Notes
*Asterochloris sp*. v1.0	35232	scaffold_00088:18492-25512 [JGI Genome Portal]	Glycolytic enolase
*Chlamydomonas reinhardtii*	136652	Cre12.g513200.t1.2; chromosome_12: 3521433-3525524 [Phytozome v9.1]	Glycolytic enolase
*Chlamydomonas reinhardtii*	NA	g393; chromosome_1: 2781663–2787514 [Phytozome v9.1]	Multi-functional enolase
*Chlorella variabilis* NC64A	136652	scaffold_17:245647-250804 [JGI Genome Portal]	Glycolytic enolase; Gaps in genomic sequence
*Coccomyxa subelipsoidea*	38308	fgenesh1_pm.19_#_130; scaffold_19: 1328456–1334257 [Phytozome v9.1]	Glycolytic enolase
*Coccomyxa subelipsoidea C-169*	35576	fgenesh1_pm.3_#_251; scaffold_3: 2788580–2794381 [Phytozome v9.1]	Glycolytic enolase
*Coccomyxa subelipsoidea C-169*	NA	estExt_Genewise1Plus.C_20589; scaffold_2: 2932335–2935743 [Phytozome v9.1]	Multi-functional enolase
*Micromonas pusilla* CCMP1545	122580	scaffold_2: 722515–724505 [Phytozome v9.1]	Glycolytic enolase
*Micromonas pusilla* RCC299	107587	Chr_01: 1470509–1472948 [Phytozome v9.1]	Glycolytic enolase
*Ostreococcus lucimarinus*	28765	Chr_1: 271278–273263 [Phytozome v9.1]	Glycolytic enolase
*Ostreococcus tauri* v2.0	27349	Chr_01.0001:228132-230031 [JGI Genome Portal]	Glycolytic enolase
*Volvox carteri*	79991	Vocar20013958m.g; scaffold_6: 3504767–3509879 [Phytozome v9.1]	Glycolytic enolase
*Volvox carteri*	42159	Vocar20006493m.g; scaffold_7: 3486914–3491916 [Phytozome v9.1]	Multi-functional enolase
*Dunaliella salina* CCAP19/18	-	GenBank accession number KM008612	Glycolytic enolase
*Dunaliella salina* CCAP19/18	-	GenBank accession number KM008613	Multi-functional enolase

**Figure 3:**
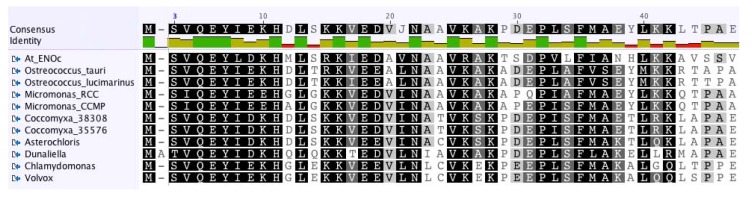
Shown is an overview of a MUSCLE [67] alignment (Score Matrix = Blossum62) of the N-terminus of enolase protein sequences from selected green algae and of ENOc of the higher plant *A*. *thaliana*. The *P*. *provasolii* enolase proteins were not included in this Figure, because the N-terminal sequences are not known. Color code: Black = 100% similar; Dark Gray = 80%–100% similar; Light Gray = 60%–80% similar; White = less than 60% similar.

**Figure 4:**
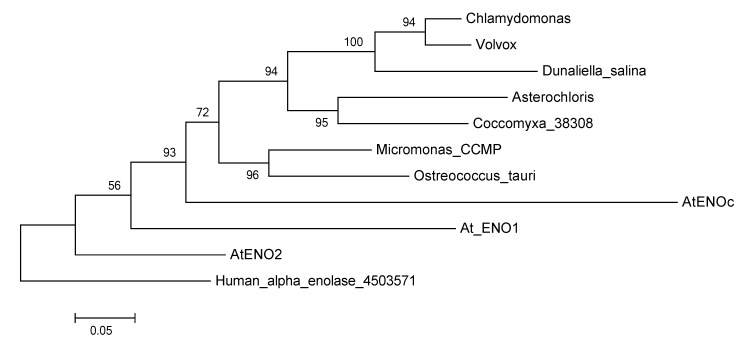
Protein Maximum Likelihood phylogeny reconstruction based on 500 bootstraps of the glycolytic enolase genes including several green algae and the three different glycolytic enolase forms from *A*. *thaliana*. Tree was rooted with the human alpha enolase. The multi-functional enolases were not included in this phylogeny, because they are too different from the glycolytic enolases and resulted in very low support values.

To learn more about the possible function and cellular location of the green algal enolase(s), we compared the algal enolase protein sequences with the known enolase proteins of the higher plant *Arabidopsis thaliana*–as well as the one found in the human genome. First, we aligned the green algal sequences together with the four sequences obtained from the higher plant *A*. *thaliana* ([51]-ENO1, ENO2, ENOc, multi-functional ENO) and with the human alpha-enolase. As expected from the results of the previous study by [59], the cytosolic enolase (ENOc) of *A*. *thaliana* showed closest homology to the green algal glycolytic enolases (Figure 3). As presently more full-length green algal enolase sequences are available than were for previous studies, we performed a new phylogenetic analysis. The recovered glycolytic enolase phylogeny followed the basic taxonomy of the green algae included in our study (Figure 4).

It was noteworthy that the cytosolic enolase from *A*. *thaliana* and all green algal enolases, for which complete sequences were available, had a similar N-terminal extension (Figure 3) indicating that the investigated genes are paralogous. The N-terminal extension consisted of about 45 amino acids and was almost as long as the known transit peptide sequence of the plastid-localized ENO1 of *A*. *thaliana*. However, the N-terminal extension of the cytosolic form of enolase from *A*. *thaliana* and the green algal sequences had no similarity to the transit peptide of the plastidal form of enolase from *A*. *thaliana*. In addition, using multiple prediction programs (including PredAlgo [68]) no transit peptide was predicted for the enolase proteins containing the N-terminal extension (results not shown). Our general comparison of enolase proteins from green algae to the plastidic and cytosolic forms of enolase of *A*. *thaliana* suggested that the algal enolase enzymes perform similar functions as the cytosolic enolase in *A*. *thaliana* and that the algal enzymes are also located to the cytosol and/or the nucleus. Our hypothesis that the enolase in green algae may be absent from the plastids is in line with previous results for *C*. *reinhardtii*, where the enolase substrate 2-phosphoglycerate [28], the enolase activity [27], or the enolase protein [26] could not be detected in the plastid of this model alga.

**Figure 5:**
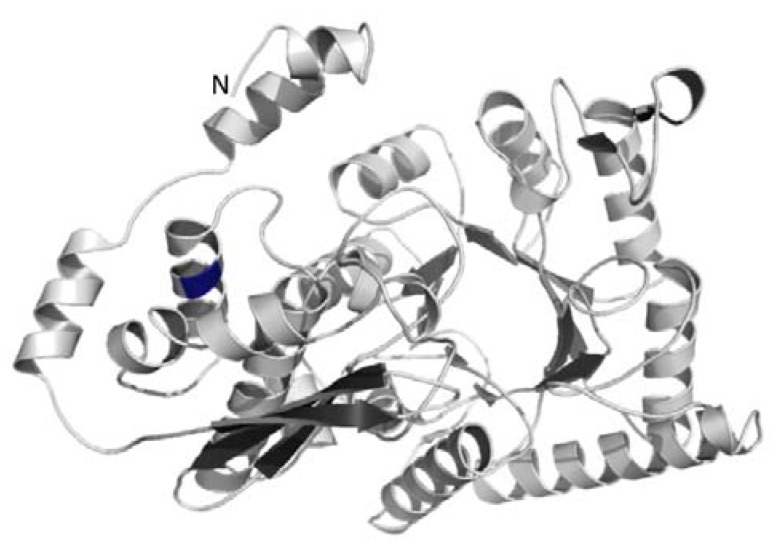
Shown is a ribbon representation of the putative tertiary structure of a monomer of the N-terminally extended enolase of the green alga *C*. *reinhardtii*. The N-terminal β-sheet with 3 strands and the C-terminal β-sheet with enolase active site are shown in dark grey. The location of the potential alternative translation start site Met136 is shown in blue.

Thus far, the function of the N-terminal extension of the AtENOc is unknown. Based on comparison with other enolase enzymes such as human alpha-enolase, all catalytic residues and the residues involved in dimer formation are contained outside the N-terminal extension. To potentially find out more about the function of the highly conserved N-terminal extension also present in the proteins of green algae, we chose as a representative of an enolase containing the N-terminal extension the protein sequence from *C*. *reinhardtii* for modeling of the tertiary structure. Modeling was done by submission of the *C*. *reinhardtii* enolase protein sequence to ITASSER [69]. The five model structures obtained from ITASSER were based on enolase crystal structures from human and animal systems. However, none of the available crystal structures from animals/human contained the N-terminal extension of about 45 amino acids. Therefore, the different 3D-structure models for enolase of *C*. *reinhardtii* differed mainly in the overall arrangement of the three alpha helices contained in the 45 amino acid N-terminal extension. Figure 5 shows the best evaluating model obtained for *C*. *reinhardtii* enolase from ITASSER. The 3D-model did not reveal any specific function for the N-terminus, but it may be speculated that the additional sequence could impact polymerization of the algal enolase into homo-dimers, thus playing a regulatory role. More extended structural studies would be needed to demonstrate the role of the N-terminus of the algal glycolytic enolase. Such detailed studies were beyond the scope of this manuscript.

Further investigation of the sequence of the *A*. *thaliana* cytosolic enolase and the green algal enolase proteins also revealed that there exists a conserved methionine either in position 136 or 137. This conserved methionine is at the same position as the described alternative translational initiation site for the AtENO2/AtLOS2 resulting in the truncated translation product AtMBP-1 [45]. Our prediction of the enolase structure of *C*. *reinhardtii* revealed that the conserved alternate translation start position, which codes for the methionine in the full-length enolase, is located in a highly conserved alpha-helix (Figure 5). The highly conserved structure also found in the algal enolase might indicate that a truncated MBP-1-like protein with transcriptional suppression activity could also exist in the green algae. However, an investigation into confirming this hypothesis is beyond the scope of our manuscript.

Due to the similarity to the glycolytic enolase, we also included the DEP1 of *A*. *thaliana* for our homology searches in green algal genomes. Based on homology, we were able to identify a gene coding for the multi-functional DEP1 in several green algae (Table 1). This result was not unexpected, as the methionine salvage pathway is widely distributed among eukaryotes. Although DEP1 is not known to function as a phosphopyruvate hydratase, it will be necessary to perform biochemical analyses of the algal DEP1 to test if this enzyme is able to perform the glycolytic enolase reaction for providing evidence that the glycolytic enolase activity cannot occur in algal plastids.

Our findings regarding the existence of one glycolytic enolase in several algal genomes with the glycolytic enolase most likely localized to the cytosol only supports our hypothesis raised above that the enolase could be a possible bottleneck in carbon partitioning in green algae (Figure 2). That the glycolytic enolase could be important in adjusting cellular carbon partitioning in response to environmental changes was indicated for example in the halophilic green alga *D*. *salina*. In *D*. *salina*, it was shown that during exposure to hyperosmotic salt conditions the enolase was transiently down-regulated [50,70]. Such down-regulation of the glycolytic enolase could result in “pushing” carbon to glycerol-3-phosphate (Figure 2) and glycerol, which is the osmotic substance in *D*. *salina*. Another recent study on metabolism of cells of *C*. *reinhardtii* shifted from low-light to a higher-still limiting-light intensity showed a resulting small increase in the transcript level of enolase, but no significant change in protein abundance of enolase [71]. No data were presented on changes in the activity of the enolase under the conditions investigated, but observed were increased starch contents in cells, decreased chlorophyll levels, and an increased carbon flux into lipid biosynthesis [71]. At this time, we do not have any information on how the enolase activity might be regulated in green algae. Speculating that the enolase activity was not altered, the results from the study with *C*. *reinhardtii* [71] would fit into our model as presented in Figure 2. With more carbon fixed under higher irradiance, but no change in the activity of the enolase, our model would predict a bottleneck at the level of enolase, thus, resulting in accumulation of starch, but no change in the amount of carbon provided to pyruvate. At the level of pyruvate, glycerolipids compete for carbon with isoprenoids: thus our model would predict that with decreasing levels of chlorophyll, which contains isoprene units, under higher limiting irradiance levels carbon would be re-directed to glycerolipid biosynthesis. In short, our model on carbon partitioning in green algal cells agrees with the results reported for *C*. *reinhardtii* [71].

In summary, our comparative study of enolases from sequenced genomes of green algae showed that most of the investigated green algae only had one gene–for the glycolytic enolase–in their genome. This indicates that the enolase exists either as monomers or as homo-multimers. The green algal enolase sequence is very similar to the cytosolic ENOc of *A*. *thaliana*, also including an N-terminal extension of unknown function that is different from a signal peptide. The high similarity to AtENOc strongly suggests that the green algal enolase is also only localized to the cytosol making it a potential bottleneck in carbon partitioning of algal cells (Figure 2). Further, existence of an alternative translation initiation site allows the speculation that similar to human and plants, the MBP-1 transcription suppressor might exist in green algae.

## 3. Experimental Section

### 3.1. Comparative Genomics

For analysis of genomes for presence of genes coding for the enolase and the multi-functional enolase in green algae the web interface of the portal of the US Department of Energy Joint Genome Institute [72] with the links to individual green algal genomes was used. Alternatively, Phytozome v9.1 [73] was used to retrieve sequences.

### 3.2. Phylogenetic Analysis

Protein sequences were downloaded from Phytozome v9.1, with *Chlamydomonas reinhardtii* v5.5, as well as the National Center for Biotechnology Information (NCBI). They were then aligned using MUSCLE [67] in Geneious [74] and MEGA5.2.2. The muscle alignment was then used in MEGA5.2.2 to create a phylogenetic reconstruction using Maximum Likelihood, with 500 bootstraps, and the Jones-Taylor-Thorton (JTT) model with uniform rates of substitution.

### 3.3. Structural Modeling of the Enolase Enzyme

The protein sequence of the Enolase of *C*. *reinhardtii* was submitted to the on-line I-TASSER server [69] for protein structure and function predictions.

## 4. Conclusions

Our results of the screen of green algal species genomes for existence of glycolytic enolase genes revealed that, in the investigated species, one or more gene copies exist coding for the enolase. However, for several green algae, the glycolytic enolase is coded for by only one gene. This situation is in contrast to higher plants such as *A*. *thaliana*, providing evidence that no differential regulation exists in these algae. Moreover, presence of only one gene coding for a protein similar to the cytosol-localized AtENOc suggests that none of the investigated green algae contains a plastid-localized enolase enzyme. In line with previous results for *C*. *reinhardtii* [26,27,28,29], we propose that in green algae the glycolytic enolase is only active in the cytosol resulting in a specific pattern of carbon flow following fixation (Figure 2), involving export of carbon from the plastid and then re-import for pyruvate generation. Based on our genomic analysis of multiple green algal species, this specific path of carbon processing with an essential reaction only localized to the cytosol appears to be a common pattern within the green algal lineage.

In comparison to multi-cellular vascular plants and as a consequence to the only cytosol-localized enolase, to regulate carbon partitioning in response to environmental changes in unicellular green algae the enolase may have to be more tightly controlled than in higher plants. Consequently, open questions to investigate for green algae are (1) how expression and activity of the enolase are regulated and (2) if that regulation could be correlated to changes in carbon partitioning such as flow of carbon to isoprenoids and glycerolipids.

We predict that manipulation of enolase expression and activity-possibly in combination with altering the expression/activity of the cytosolic phosphoglycerate mutase (EC 5.4.2.11 or EC 5.4.2.12)-will have significant impacts on metabolic engineering of green algae. Depending on which substrates desired product(s) would require and where in the cell the product(s) should be made, carbon flux through PEP made by the enolase may be crucial for directing carbon towards the product(s). Moreover, with the enolase and possibly other enzymes of the lower glycolysis pathway [26,27,28] only present in the cytosol, opportunities to alter metabolic flux through introduction of this pathway capability into the plastid of green algae may open up unique avenues for novel metabolic engineering purposes.

Based on structure comparison to other enolases from humans and animals, the present N-terminal region does not perform catalytic function within the enolase enzyme of green algae. This led us to hypothesize that this N-terminus could play a role in regulation of the activity of the enolase enzyme.
